# Medicinal cannabis: knowledge, beliefs, and attitudes of Colombian psychiatrists

**DOI:** 10.1186/s42238-021-00083-z

**Published:** 2021-07-05

**Authors:** Juan Manuel Orjuela-Rojas, Xiomara García Orjuela, Sabina Ocampo Serna

**Affiliations:** 1 Pontificia Universidad Javeriana, Bogotá, Colombia; 2 Universidad Nacional de Colombia, Bogotá, Colombia

**Keywords:** Cannabis, Psychiatry, Medical marijuana, Colombia

## Abstract

**Background:**

The use of cannabinoids in mental health has gained strength in recent years due to emerging scientific evidence and the lifting of prohibitionist laws that prevailed for years in many countries, including Colombia. This study describes the results of a survey of Colombian psychiatrists on some aspects of medicinal cannabis, such as attitudes towards its potential use, perceived knowledge, and beliefs surrounding its regulation and safety.

**Methods:**

We conducted a cross-sectional survey of 145 psychiatrists in 14 territories of Colombia between November 2019 and July 2020. The survey consisted of 28 items on topics related to medicinal cannabis, including attitudes and clinical experience (4 items), perceived knowledge (4 items), indications for use in psychiatric pathologies (6 items), indications for use in nonpsychiatric pathologies (8 items), and concerns and awareness about safety and efficacy (6 items). The results were summarized using descriptive statistics. In addition, possible associations among variables were examined using Fisher’s exact test.

**Results:**

Eighty-two percent of the psychiatrists agreed that medical cannabis should be available for different medical conditions, and 73.1% stated that they wanted to be able to prescribe it. However, 66.2% said they did not know how to help their patients legally access it, and only 25% understood the legal status of medicinal cannabis in the country. The mental health indications that received the highest approval levels for cannabis use were insomnia (35.2%), anxiety disorders (29%), and agitation in dementia (18.6%). The greatest disapproval of cannabis use was indicated for schizophrenia, with 66.9%. The most approved nonpsychiatric medical conditions were cancer-related chronic pain (87.6%), chemotherapy-related nausea and vomiting (78.6%), and chronic pain not associated with cancer (72.4%). Multinomial stepwise logistic regression analysis showed that female psychiatrists who did not agree with MC to treat psychiatric symptoms were more likely to agree with non-psychiatric use.

**Conclusions:**

Our results showed that this sample of Colombian psychiatrists have a favorable attitude towards the prescription of medicinal cannabis; however, there is a serious lack of knowledge of the legal status of medicinal cannabis in the country and the methods through which patients can gain access to government-regulated products. Most of them approve the use of MC for nonpsychiatric conditions and, in general, disapprove of its use in mental illnesses. They generally consider medicinal cannabis as a safe treatment compared to other psychotropic drugs and medications with potential risk of dependence, such as opioids and/or benzodiazepines.

**Supplementary Information:**

The online version contains supplementary material available at 10.1186/s42238-021-00083-z.

## Background

In August 2017, the National Government of Colombia finalized the regulatory process for the manufacture, use of seeds, and cultivation of cannabis for medicinal and scientific purposes. As a result, the medicinal cannabis (MC) industry has seen growth and development in different regions of the country, generating fertile ground for the legal use of the plant for multiple physical and mental conditions (Arias et al., 2021). As recently as March 2020, the Colombian National Institute of Drug and Food Surveillance (INVIMA) issued the first approval of good practices for the preparation of cannabis-based products, marking the beginning of the MC era in Colombia (Ledezma-Morales et al., 2020). To date, Colombia has very few legal cannabis-based pharmaceutical products; therefore, access for patients has been limited (Ledezma-Morales et al., 2020).

According to INVIMA, medicinal cannabis corresponds to the use of the flower of the Cannabis genus plant, its preparations, or its active principles, called cannabinoids—among them tetrahydrocannabinol (THC), cannabidiol (CBD) and cannabinol (CBN)—as therapy to treat some diseases or relieve symptoms (INVIMA, 2021).

There are numerous documented barriers to the adoption of MC in the medical community. One of them is the lack of knowledge among the medical profession regarding the doses, indications, and adverse effects of cannabis-based preparations (Alexander, 2020). In part, this is due to the limited importance that medical schools place on MC-related content (Paut Kusturica et al., 2019) and its possible health benefits (Karanges et al., 2018). Another barrier is that most doctors feel safer prescribing evidence-supported drugs, and in the case of MC, especially as it relates to the area of mental health, the evidence is limited and of low quality (Sarris et al., 2020). Finally, there are some barriers for its prescription by psychiatrists in particular the lack of regulatory approval for their prescription and the relatively scant literature of scientific and well-controlled studies of the safety and efficacy of MC for mental diseases. Furthermore, psychiatrists are more familiar with the harmful effects of cannabis on mental health, particularly with recreational use (due to high concentrations of THC) and its deleterious outcomes such as psychosis, anxiety, and depression with long-term use (Gobbi et al., 2019; Moore et al., 2007).

However, with the discovery of the endocannabinoid system in the 1990s (Friedman & Sirven, 2017; Matsuda et al., 1990) and the relaxation of prohibitionist laws regarding cannabinoids, like THC or CBD, multiple countries have approved its medical use for different conditions, such as chronic pain, refractory epilepsy, spasticity associated with multiple sclerosis and nausea, and vomiting associated with chemotherapy, for which the evidence base is greater (Fraguas-Sánchez & Torres-Suárez, 2018). Limited evidence in the field of mental health leans towards using cannabinoids to treat social anxiety disorders (Bergamaschi et al., 2011), sleep disorders (Shannon, 2019), mood disorders associated with chronic pain (e.g., fibromyalgia) (Habib & Artul, 2018), posttraumatic stress disorder (PTSD) (Roitman et al., 2014), psychosis in Parkinson’s disease (Zuardi et al., 2009), and refractory schizophrenia (McGuire et al., 2018) and to manage behavioral symptoms in dementias (Woodward et al., 2014).

A survey of 62 primary care providers conducted in the Minnesota-based healthcare system obtained information about provider characteristics, attitudes, and beliefs about MC. The participants considered that MC was useful for treating medical conditions such as cancer, terminal illnesses, and intractable pain. Although the survey did not emphasize psychiatric symptoms and did not include mental health workers, the level of approval for use was 45% for anxiety, 27% for sleep disorders and 12% for depression (Philpot et al., 2019). Another study on the perception of MC among medical students in Serbia only considered nonpsychiatric medical indications for its use and took psychiatric symptoms such as anxiety, depression, sleep disturbances, and psychosis only as adverse effects (Paut Kusturica et al., 2019). Another survey of 640 Australian general practitioners revealed strong support for the use of MC for cancer pain, palliative care and epilepsy but low support for its use for depression and anxiety (Karanges et al., 2018). Finally, the only survey of psychiatrists (and residents of psychiatry) conducted to date in Australia showed that of the 88 participants, the majority believed there was evidence for the use of CBD and THC for the treatment of childhood epilepsy, chronic pain and nausea, and vomiting. However, they were concerned about the adverse effects of MC and its risk of generating psychotic symptoms, addiction, apathy, and being used recreationally (Jacobs et al., 2019).

The purpose of the following study is to determine the attitudes of Colombian psychiatrists towards the use of MC, its indications in psychiatric and non-psychiatric conditions, and perceived knowledge and beliefs about its regulation and safety. To the best of our knowledge, no similar studies have been published involving psychiatrists outside Australia (Jacobs et al., 2019).

## Methodology

Between November 2019 and July 2020, a survey was conducted with multiple psychiatrists from different cities in Colombia. A total of 150 psychiatrists were contacted; of these, 145 responded to the survey in its entirety (96% response rate). The online survey was sent to the psychiatrists prior to talks and conferences on MC in different mental health hospitals and educational institutions in the country. Psychiatric residents did not participate. Demographic data were collected, such as age, gender, city in which the participant worked, and number of years practicing as a specialist.

The survey itself consisted of 28 items on topics related to MC, including attitudes and clinical experience (4 items), perceived knowledge (4 items), indications for use in psychiatric pathologies (6 items), indications for use in nonpsychiatric pathologies (8 items), and concerns and awareness about safety and efficacy (6 items). We developed the survey taking as an example the research of Karanges et al. (2018), which had similar objectives to evaluate knowledge, attitudes, and beliefs related to MC. It was not strictly subjected to a validation process, but we did carry out a pilot prior to the formal application of the survey. The pilot included 10 physicians to determine response times, ease of understanding questions, and topics relevant to the research. According to our observations, we decided to simplify the responses to a 3-point Likert scale: agree, neutral, or disagree, and we added 1 item corresponding to schizophrenia.

Symptoms of mental disorders for which studies with cannabinoids had been performed, such as anxiety, posttraumatic stress disorder, insomnia, behavioral symptoms in dementia, schizophrenia, and depression, were included. Additionally, the survey included questions about symptoms associated with nonpsychiatric medical diseases for which the use of cannabinoids has been investigated, such as neuropathic pain, cancer-related chronic pain, chronic pain not associated with cancer, spasticity in multiple sclerosis, refractory epilepsy, cachexia, cancer (antitumor), and nausea and vomiting associated with chemotherapy. For more details, see Appendix 1.

**Fig. 1:**
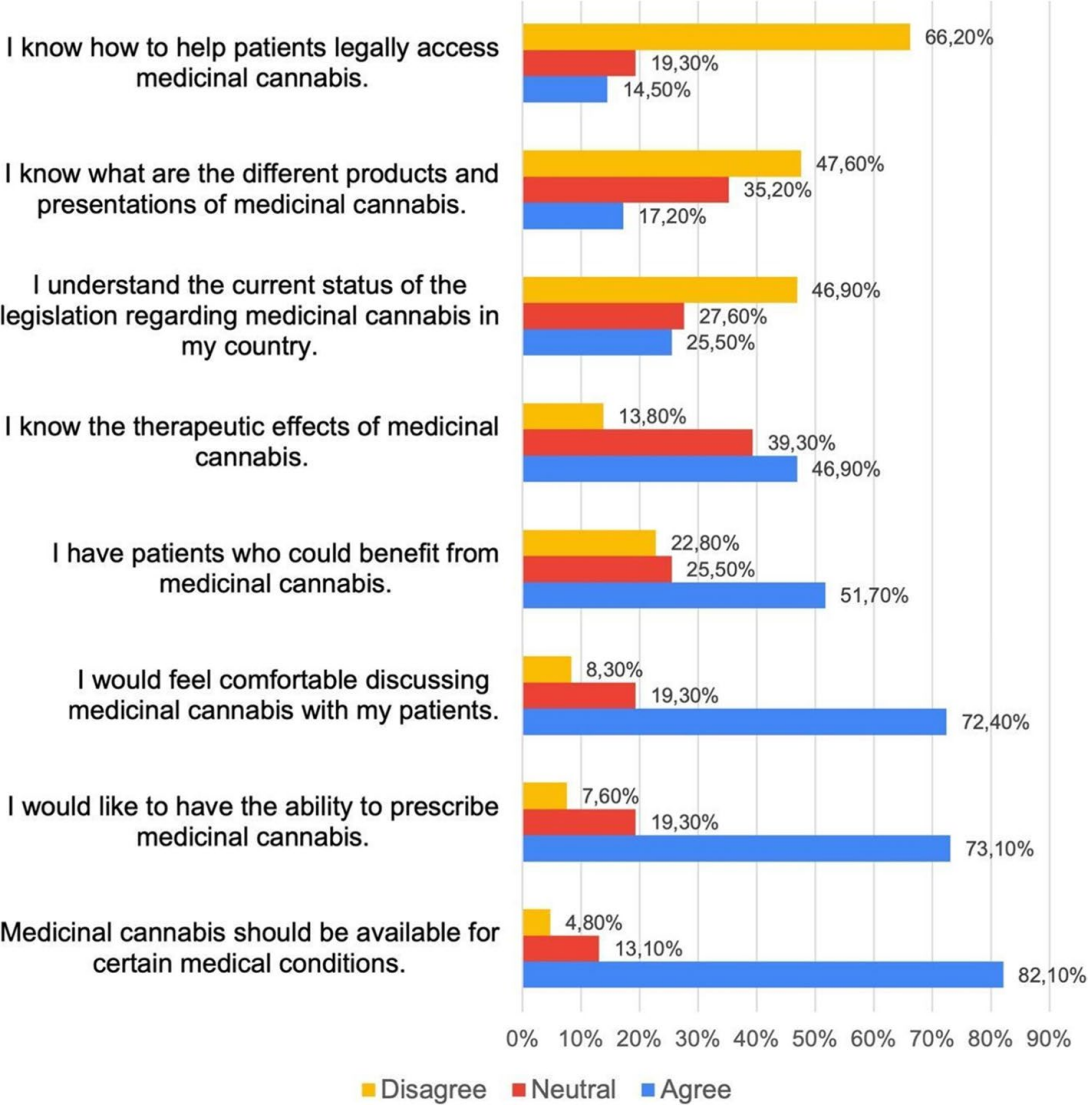
Attitudes and knowledge perception with respect to medicinal cannabis among 145 Colombian psychiatrists. The survey showed that the majority of Colombian psychiatrists believe that MC should be available for different medical conditions. However, there is a lack of knowledge of the legal status of cannabis in the country. MC, medicinal cannabis

**Fig. 2:**
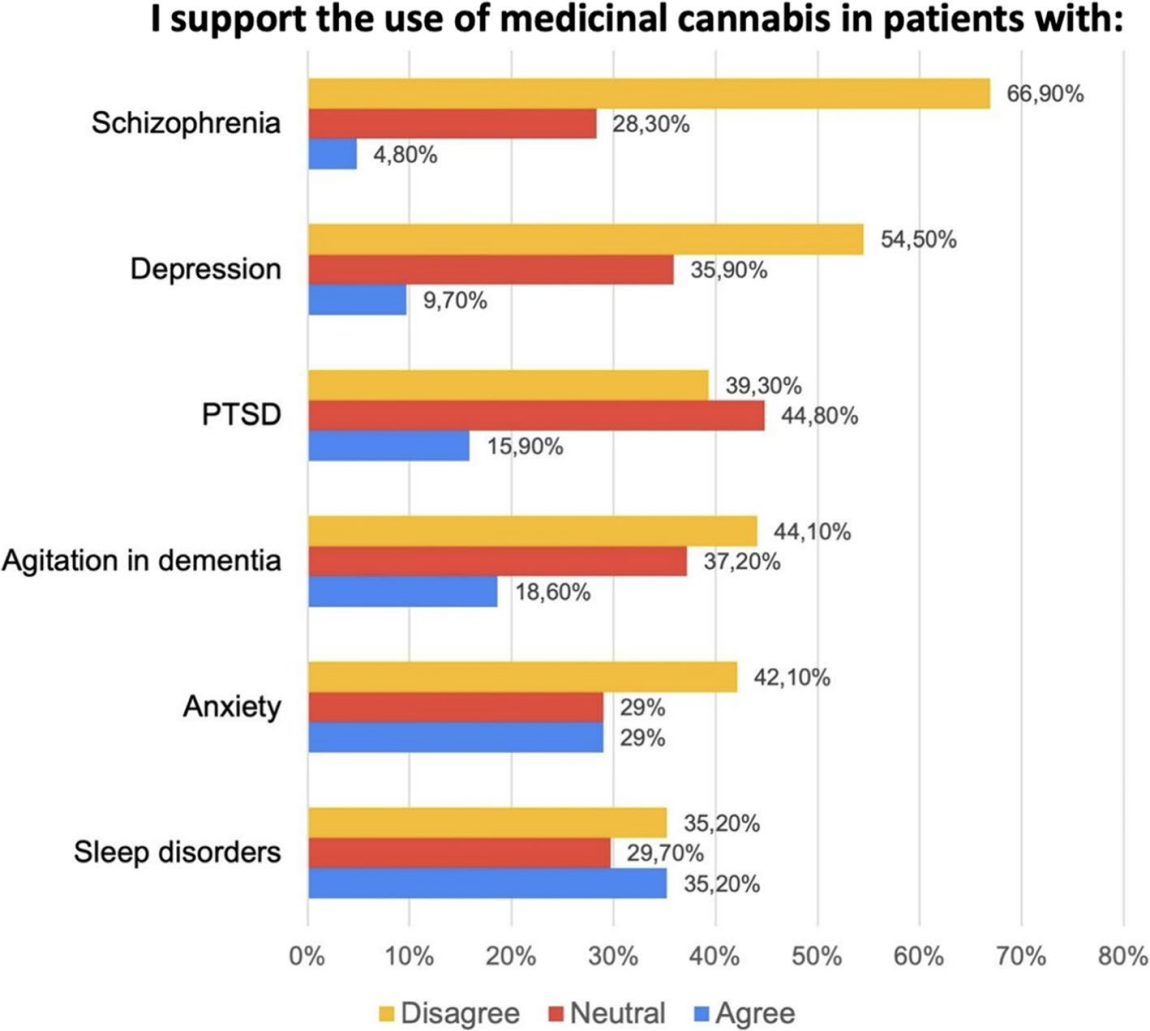
Support for use of medicinal cannabis in different mental health conditions among 145 Colombian psychiatrists. The mental health condition with the highest approval for the use of MC was sleep disorders and the one with the most disapproval was schizophrenia. MC, medicinal cannabis

**Fig. 3:**
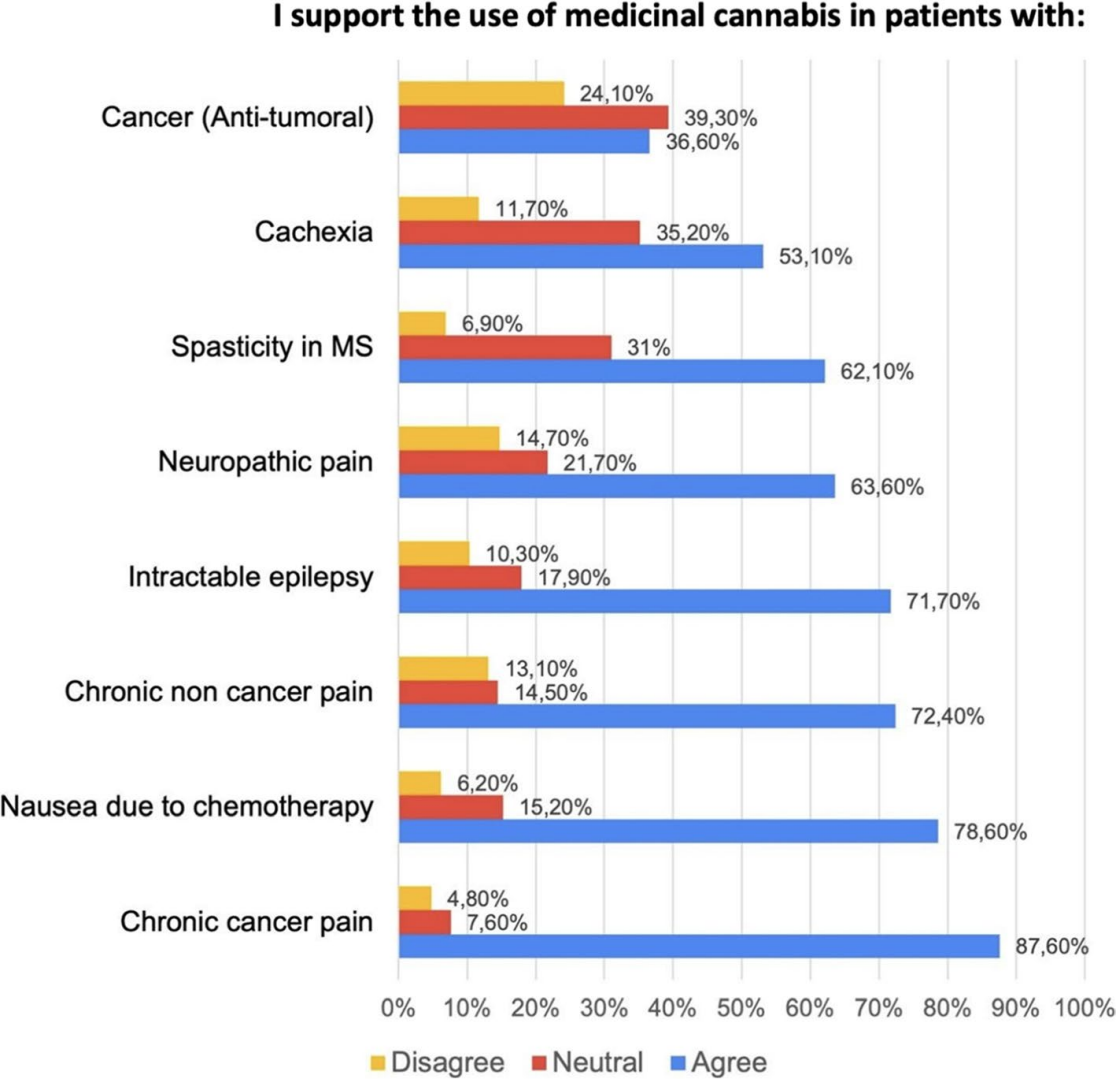
Support for use of medicinal cannabis in non-mental health conditions among 145 Colombian psychiatrists. The non-mental health condition with the highest approval for the use of MC was chronic cancer pain and the one with the most disapproval was antitumoral effect. MC, medicinal cannabis

**Fig. 4:**
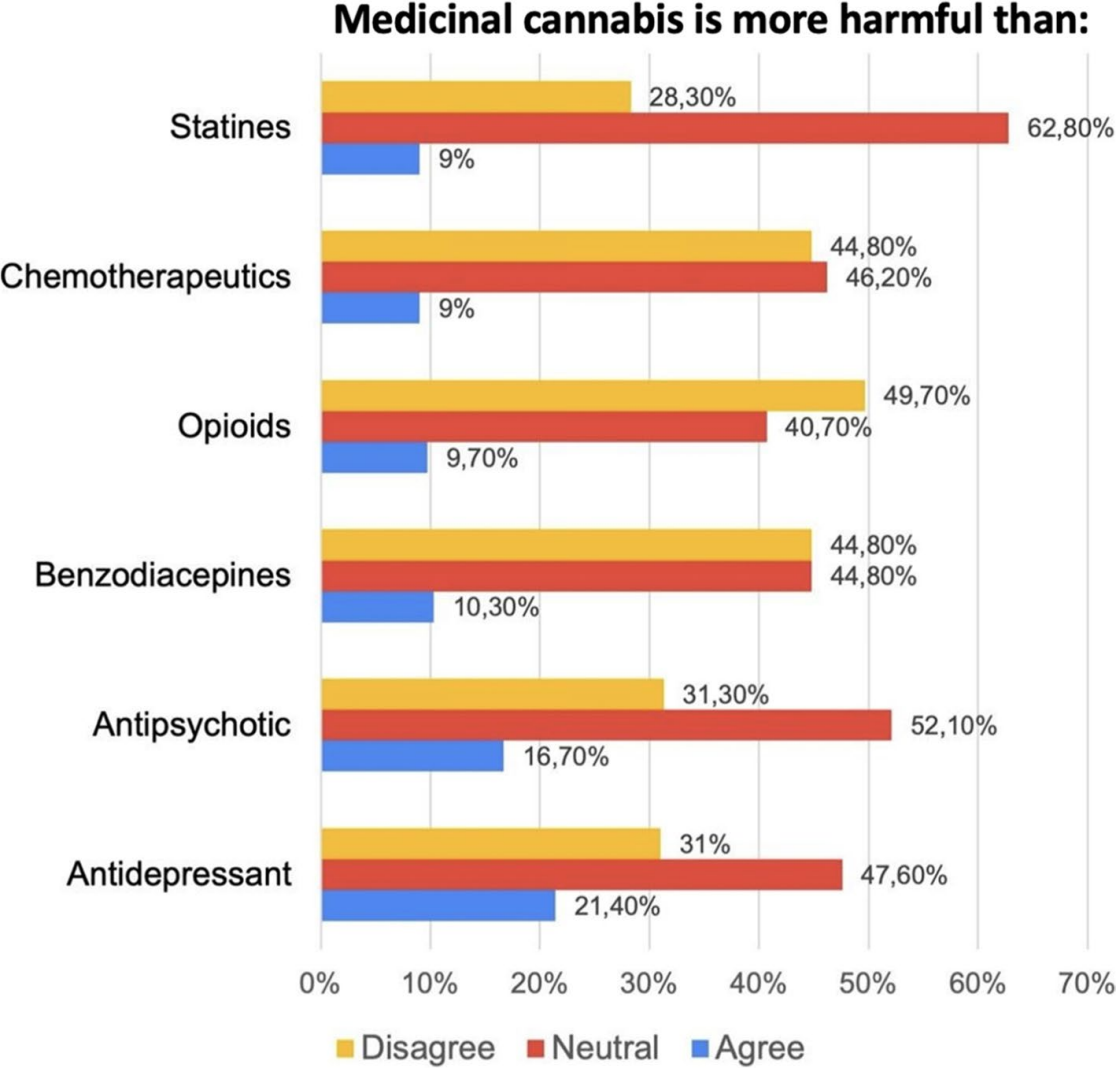
Ratings of relative hazards of medicinal cannabis compared with other prescription medicines among 145 Colombian psychiatrists. Most of the psychiatrists surveyed were neutral about or disagreed with whether cannabis was more harmful than other medications

The average survey duration was 5–10 min. The results are expressed in terms of percentages (see Figs. 1, 2, 3, and 4). This study was approved by Dexa Diab Scientific Research Ethics Committee. Before answering the survey, participants were informed that the purpose of the study was to determine knowledge and attitudes related to MC and that by entering the responses they were agreeing to be part of it.

### Statistical analysis

The results were summarized using descriptive statistics (frequency, percentage of valid responses). We used a forward selection multinomial logistic regression to estimate relative risk ratios for the likelihood of recommending MC compared to all demographic variables in the first place, and the likelihood of recommending MC to or not treat psychiatric or non-psychiatric symptoms. Confidence interval was set at 95%, *P* value < 0,05. Selection criteria for each step were performed by the software using likelihood ratio and McFadden pseudo R depending on covariate or factor. In addition, possible associations among variables were examined using Fisher’s exact test, and the results were considered significant at *p *< 0.05. IBM SPSS Statistics for Windows, V.24.0 (IBM), software was used, and graphs were created using GraphPad Prism V.9 for Windows (GraphPad Software, La Jolla, CA, USA).

## Results

### Demographic data

The demographic data are summarized in Table 1. Most of the participants were women (55.2%). The predominant age range was 35 to 55 years (51.7%), and there was a greater representation of psychiatrists located in the state/territory of Cundinamarca (44.8%). In total, doctors from 15 Colombian cities participated in the study. Most of the respondents had between 5 and 10 years of experience (42.1%).

**Table 1:** Demographic and practice characteristics of psychiatrists in this study

	*n* = 145	%
**Age**		
< 35 years	43	29.7%
35–44 years	75	51.7%
45–54 years	14	9.7%
> 55 years	13	8.9%
**Sex**		
Male	65	44.8%
Female	80	55.2%
**Years as specialist**		
< 5 years	43	29.7%
5–10 years	61	42.1%
> 10 years	41	28.3%
**State/territory**		
Amazonas	1	0.7%
Antioquia	14	9.6%
Atlántico	9	6.2%
Caldas	9	6.2%
Cundinamarca	65	44.8%
Magdalena	1	0.7%
Meta	2	1.3%
Nariño	5	3.4%
Quindío	1	0.7%
Risaralda	7	4.8%
Sucre	2	1.3%
Santander	13	8.9%
Tolima	1	0.7%
Valle	15	10.3%

Demographic results of the survey conducted in 145 Colombian psychiatrists between November 2019 and July 2020

### Attitudes and perceived knowledge

The vast majority of the psychiatrists agreed that MC should be available for different medical conditions (82.1%). More than half of the participants felt that they had patients who could benefit from the use of MC (51%), and a large majority reported wanting to be able to prescribe cannabis (73.1%). Similarly, they expressed that they would feel comfortable discussing MC with their patients (72.4%) (Fig. 1).

Overall, the MC knowledge of Colombian psychiatrists is low. Two-thirds of the respondents (66.2%) said they did not know how to help their patients legally access cannabis, and 46.9% did not understand the legal status of MC in the country. Additionally, less than half of the respondents (46%) knew the therapeutic effects of cannabis, and only 17.2% knew the therapeutic presentations of cannabis-based products.

### Indications for use in psychiatry

The results revealed that the only mental health condition for which the surveyed psychiatrists showed relatively high agreement regarding MC use was insomnia (35.2%). Despite the level of agreement, 29.7% had a neutral response, and 35.2% disagreed; therefore, 64.9% of the sample did not perceive this indication positively. The second most accepted mental health condition was anxiety disorders (29%), followed by agitation in dementia (18.6%), PTSD (15.9%), depression (9.7%), and schizophrenia (4.8%). In the case of PTSD, although the degree of disapproval was 39.3%, most of the participants were neutral (44.8%). The pathology for which MC use had the greatest disapproval was schizophrenia, with 66.9% (Fig. 2).

### Indications for nonpsychiatric use

Most of the psychiatrists approved of the use of MC for nonpsychiatric pathologies. The highest approval was for cancer-related chronic pain (87.6%), followed by nausea and vomiting associated with chemotherapy (78.6%), chronic pain not associated with cancer (72.4%), intractable epilepsy (71.7%), neuropathic pain (63.6%), spasticity in multiple sclerosis (62.1%), cachexia (53.1%), and cancer (antitumor) (36.6%) (Fig. 3).

Comparing the approval of MC use in psychiatric versus non-psychiatric symptoms, we found a statistically significant difference (*p *= 0.003). Table 2 shows the distribution of frequencies and percentages regarding the support of use of MC for these indications among participants and Table 3 shows Fisher’s exact test.

**Table 2:** Distribution of frequencies and percentages regarding the support of use of MC for psychiatric and non-psychiatric symptoms among participants

		**Support use of medicinal cannabis non-psychiatric symptoms**		**Total**
		No	Yes	
**Support use of medicinal cannabis for psychiatric symptoms**	No	9 (7%)	66 (45%)	75 (52%)
	Yes	0	70 (48%)	70 (48%)
**Total**		9 (7%)	136 (93%)	145 (100%)

Despite 93% of psychiatrists support MC use in nonpsychiatric symptoms, only 48% support its use to treat psychiatric symptomatology. *MC* medicinal cannabis

**Table 3:** Chi-square and Fisher’s exact test values resulting from the cross tab analysis of recommendation of MC use for psychiatric and non-psychiatric symptoms among psychiatrists. As long as one of the values in the cross tab is below 5, Fisher’s exact test is the preferred tool

	**Value**	**Asymptotic significance**	**Bilateral exact significance**	**Unilateral exact significance**
Pearson’s chi-square	8956	0.003		
Fisher’s exact test			0.003	0.002
Linear by linear association	8894	0.003		
Valid cases	145			

### Perception of the safety and adverse effects of medicinal cannabis

In all cases, the participants felt that MC was less harmful than most of the drugs named in the study. For example, only 10.3% agreed that cannabis was more harmful than benzodiazepines, and only 9.7% agreed that MC was more harmful than opioids. For other drugs, such as antipsychotics, chemotherapeutics, and statins, the trend was similar (Fig. 4); however, the participants perceived cannabis as having higher risks than antidepressants, with 21.4% considering cannabis more harmful. It is important to note that the predominant response was neutral, reaching values of up to 62.8% for statins and 51.7% for antipsychotics.

Interestingly, female psychiatrists who did not agree with the use of medical cannabis to treat mental symptoms were more likely to support use of medical cannabis for non-psychiatric pathologies (*p *= 0,006). No other statistically significant results were documented throughout the multinomial regression analysis.

## Discussion

This study aimed to evaluate the perceived knowledge, beliefs, and attitudes of Colombian psychiatrists regarding the use of MC. To our knowledge, this is the first study of these characteristics conducted outside Australia and focusing only on specialists in psychiatry. The survey showed that the majority of the sample of Colombian psychiatrists believe that MC should be available for different medical conditions and expressed a desire to be able to prescribe it. In addition, they were open to starting a dialogue with their patients on issues related to MC. However, there is a serious lack of knowledge of the legal status of cannabis in the country and the methods through which patients can gain access to government-regulated products. Clarity regarding the presentations of cannabis-based products—that is, whether the drug is available in oils, dried buds, creams, and/or capsules—is lacking. This situation opens the doors for optimizing education and training in MC in mental health workers. In different countries of the world, including Colombia, there are many myths and misleading advertising of MC that generates false expectations in patients, for example, that it cures cancer (Shi et al., 2019). Many patients consult their physicians with that expectation, and it is the medical community that should educate the general public regarding the real benefits and risks of MC. For this reason, we believe that doctors (including psychiatrists) in Colombia should optimize their knowledge related to MC to offer their patients a realistic and ethical vision of its use. Previous surveys have reported a strong desire among health workers to receive continuing medical education on MC given the global expansion of its use (Kondrad & Reid, 2013).

Regarding indications for MC use in psychiatry, the condition with the highest approval was insomnia (35%). Since the remaining responses were neutral or disapproving, this indicates that most of the physicians surveyed would have concerns about using cannabinoids for insomnia, although there is currently moderate evidence supporting the use of MC in insomnia secondary to chronic neuropathic pain, fibromyalgia, and multiple sclerosis (National Academy of Sciences, 2017). Anxiety received the second-highest approval rate of 29%, a relatively low figure given the growing, but limited, evidence supporting the use of CBD for social anxiety disorder (Bergamaschi et al., 2011). The condition that generated the greatest disapproval was schizophrenia; this finding is completely reasonable since most psychiatrists associate cannabis use with a risk of psychosis, which is a real risk with nonmedical or recreational use, which tends to involve high doses of THC, especially among young people (Moore et al., 2007). However, recent studies have shown that CBD acts as an excellent adjunctive antipsychotic for the treatment of patients with refractory schizophrenia (Leweke et al., 2012; McGuire et al., 2018) and psychosis associated with Parkinson’s disease (Zuardi et al., 2009).

An interesting finding of the survey was that the majority of the psychiatrists agreed with the use of MC for nonpsychiatric medical conditions, with cancer-related chronic pain receiving the highest approval with 87.6%, a figure much higher than the highest approval rate for a psychiatric symptom, which was for insomnia (35.2%). When comparing the approval of MC use in psychiatric versus nonpsychiatric symptoms, we found a statistically significant difference (*p *= 0.003). This could indicate that psychiatrists feel more comfortable with the use of MC and perceive it as safer and more effective for specialties other than their own, such as pain medicine and palliative care. This finding was similar to that of other surveys of perceptions and knowledge regarding MC among medical psychiatrists and nonpsychiatrists; in these surveys, cannabinoids were better accepted for the treatment of nonpsychiatric medical diseases, and they had much lower approval for conditions such as insomnia, PTSD, anxiety, and depression (Jacobs et al., 2019; Karanges et al., 2018).

Regarding perceived safety and adverse effects, most psychiatrists were neutral about or disagreed with whether cannabis was more harmful than other medications. In fact, it was viewed as much safer than opioids. This perception is interesting and suggests that the old negative and even harmful connotations of cannabis in the field of mental health (Rey, 2002) could be changing with new and emerging safety and efficacy research (Hoch et al., 2019) and that, therefore, although psychiatrists are not inclined to use it for mental disorders at present, they are beginning to consider it a safe treatment compared to other psychotropic drugs and medications with potential risk of dependence, such as opioids and/or benzodiazepines.

There were several limitations of this study. The first is that the majority of the psychiatrists surveyed were located in Cundinamarca, particularly in Bogotá, the capital, where most of the psychiatrists in the country are concentrated. This could generate a selection bias, since it is likely that many of the respondents probably had similar academic background regarding MC. Another limitation is the sample size, which, although significant, represents only 12% of the professionals, taking into account that in Colombia, there are approximately 1.8 to 2.5 psychiatrists per 100,000 inhabitants, that is, about 1200 psychiatrists (The World Bank, 2020; Universidad de Antioquia, 2021). In future studies, surveys could be conducted in which indications for THC and CBD are considered separately. The percentage of women and young people (under 44 years) who responded to our survey was disproportionate, which is consistent with the general profile of people who respond to medical surveys (Cull et al., 2005) but may not necessarily represent the collective thought of the psychiatrists. The study also did not record information related to the work sector of psychiatrists, whether public or private. Finally, our findings cannot be generalized to other countries in Latin America, the USA, or Canada, among others, because access to MC and the available products and legal frameworks differ among countries. It is important to clarify that this research is merely descriptive and is not intended to promote sales of any pharmaceutical company.

## Conclusions

Our results showed that this sample of Colombian psychiatrists have a favorable attitude towards the prescription of medicinal cannabis; however, there is a serious lack of knowledge of the legal status of MC in the country and the methods through which patients can gain access to government-regulated products. Most of them approve the use of MC for nonpsychiatric conditions and, in general, disapprove of its use in mental illnesses. They generally consider MC as a safe treatment compared to other psychotropic drugs and medications with potential risk of dependence, such as opioids and/or benzodiazepines.

## Supplementary Information

**Additional file 1.**

## Data Availability

The datasets used and/or analyzed during the current study are available from the corresponding author on reasonable request.

## Abbreviations

CBD: Cannabidiol
MC: Medicinal cannabis
PTSD: Posttraumatic stress disorder
THC: Tetrahydrocannabinol
