# Supplementary material for: Medicinal cannabis: knowledge, beliefs, and attitudes of Colombian psychiatrists
Source: J Cannabis Res. 2021 Jul 5;3:26. doi: 10.1186/s42238-021-00083-z (PMC8259442; doi:10.1186/s42238-021-00083-z)
Supplement: Supplementary file 1 — Additional file 1. [file 42238_2021_83_MOESM1_ESM.pdf]

Medicinal Cannabis Survey					
This survey it's oriented to determine your knowledge and attitudes towards medicinal cannabis use (therapy based on cannabis plant, its preparations, or active principles, called cannabinoids, including tetrahydrocannabinol (THC), cannabidiol (CBD) and cannabinol (CBN) - among others, as therapy to treat some diseases or relieve symptoms). By entering your answers, you agree to be part of this study.					
e-mail address					
Select your age group (years)	<35	35–44	45–54	55–64	>65
Gender	Female		Male		
In which city do you work?					
How many years have you been practicing as a specialist?	<5 years	5 – 10 years	>10 years		

	Agree	Neutral	Disagree
Medicinal cannabis should be available for certain medical conditions			
I have patients who could benefit from medicinal cannabis			
I would like to have the ability to prescribe medicinal cannabis			
I would feel comfortable discussing medicinal cannabis with my patients			
I know how to help patients legally access medicinal cannabis			
I understand the current status of the legislation regarding medicinal cannabis in my country			
I know the therapeutic effects of medicinal cannabis			
I know what the different products and presentations of medicinal cannabis are			

I support the use of medicinal cannabis in:	Agree	Neutral	Disagree
Post-traumatic stress disorder			
Anxiety			
Sleep disorders			
Depression			
Schizophrenia			
Agitation in dementia			
Chronic cancer pain			
Intractable epilepsy			
Nausea due to chemotherapy			
Spasticity in multiple sclerosis			
Cachexia			
Cancer (Anti-tumoral)			
Neuropathic pain			
Chronic non-cancer pain			

Medicinal cannabis is more harmful than	Agree	Neutral	Disagree
Antidepressants			
Antipsychotic			
Benzodiazepine			
Opioids			
Chemotherapy			
Statins			
